# Test for Purity of Cod-Liver Oil

**Published:** 1855-11

**Authors:** 


Test for Purity of Cod-Liver Oil.—M. Berthé, as the result of numerous researches, states that when we let a drop of concentrated sulphuric acid fall on some drops of pure cod-liver oil contained in a glass plate, placed on a sheet of white paper, an aureola of the most beautiful violet is formed, which soon passes into a crimson. After a few minutes the mixture becomes brown.—(*Bulletin de l'Acad.* T. xx. 877.) *Ibid.*

